# Assessment of the characteristics of COVID-19 infection among healthcare personnel working in long-term care facilities

**DOI:** 10.1017/ash.2024.72

**Published:** 2024-05-08

**Authors:** Armaghan-e-Rehman Mansoor, Caroline A. O’Neil, David McDonald, Victoria J. Fraser, Hilary M. Babcock, Jennie H. Kwon, for the CDC Prevention Epicenters Program

**Affiliations:** 1 Division of Infectious Diseases, Department of Internal Medicine, Washington University in St. Louis, St. Louis, Missouri, USA; 2 Department of Internal Medicine, Washington University in St. Louis, St. Louis, Missouri, USA

## Abstract

Between May and June 2021, healthcare personnel at two long-term care facilities underwent SARS-CoV-2 anti-nucleocapsid immunoglobulin G testing and completed a survey on COVID-19 exposures and symptoms. Antibody positivity rate was 8.9%. Similar rates of COVID-19 exposure occurred in non-occupational and occupational settings, with high self-reported adherence to workplace infection prevention practices.

## Introduction

Healthcare personnel (HCP) in long-term care settings were significantly impacted at the beginning of the coronavirus disease 2019 (COVID-19) pandemic.^1–3^ Limitations in personal protective equipment (PPE) availability and implementation of infection control measures were some unique challenges faced by these facilities.^4^ Significant regional variation in seroprevalence has been reported among HCP in long-term care facilities, with limited reported data on specific COVID-19 exposures.^2,5^

The objective of this pilot study was to measure severe acute respiratory syndrome coronavirus-2 anti-nucleocapsid immunoglobulin G (IgG) antibody (SARS-CoV-2 anti-N IgG) positivity rate among HCP at two long-term care facilities in the St. Louis, MO metropolitan area, one year after the start of the pandemic. This antibody denotes prior COVID-19 infection and its presence is not affected by vaccination. We also examined practices related to community and workplace COVID-19 exposures, recognition of COVID-19 symptoms, PPE use, and vaccination.

## Methods

The study protocol was approved by Washington University Human Research Protection Office (IRB# 202103188). Between seven weeks in May and June 2021, HCP at a skilled nursing facility and a memory care facility in the St. Louis, MO metropolitan area were approached for enrollment through email, and via flyers placed in break rooms. All employed HCP were eligible for enrollment. There was no compensation provided for enrolled HCP. A blood specimen was obtained after enrollment and completion of informed consent to determine presence of SARS-CoV-2 anti-N immunoglobulin G (IgG) (Abbott Laboratories, Inc., Chicago, IL). Participants completed a survey with questions about demographics, job role, prior SARS-CoV-2 testing, prior symptoms consistent with COVID-19, COVID-19 vaccination, COVID-19 community and workplace exposures, and infection prevention practices. Survey data were managed using REDCap data capture tools hosted at Washington University in St. Louis.^6^ Descriptive analysis was completed using SPSS Statistics (IBM, v.27).

## Results

Seventy-nine HCP (79/220 HCPs; 35.9%) were enrolled in the study. SARS-CoV-2 anti-N IgG positivity rate in this cohort was 8.9% (7/79 HCP). Seventy-four of the enrolled participants completed the survey, including five seropositive HCP. Among the HCP who provided survey data, 82.4% were female and 51.4% reported more than ten years of healthcare experience. The majority (62.1%) had direct patient-care roles, with nurses and nurse aides comprising the largest proportion (21.6%).

All HCP reported being tested for COVID-19 at least once, and fourteen (18.9%) reported a prior positive COVID-19 polymerase chain reaction (PCR) test. Five of these fourteen HCP (35.7%) had a positive SARS-CoV-2 anti-N IgG test result. All nine HCP who reported a prior positive COVID-19 test and were seronegative stated their positive test occurred more than 3 months before study participation.

Respondents were asked whether they experienced symptoms concerning COVID-19 in the past six months. Symptoms were reported by fifty HCP (67.6%), including two of five seropositive HCP. Headaches (37.8%), fatigue (35.1%), fever/chills (27.0%), muscle or body aches (27.0%), cough (23.0%), congestion or runny nose (23.0%), and difficulty breathing (13.5%) were the most reported symptoms. When asked about perceived cause of symptoms, ten HCP (20.0%) attributed them to receipt of COVID-19 vaccine, six (12.0%) to allergies, three (6.0%) to COVID-19, and three (6.0%) to a non-COVID upper respiratory viral infection. Among HCP who reported symptoms, thirteen (35.1%) sought medical care.

When asked about known exposures to COVID-19 during the past six months, twenty-one HCP (28.4%) reported an exposure while at work, seventeen (23.0%) reported an exposure outside of work (excluding a sick household member), and thirteen (17.6%) reported a household exposure. Sixty HCP (81.1%) reported attending public events or activities in the last six months, sixty-five (87.8%) used face masks in public, and fifty-nine (79.7%) reported usually or always social distancing in public.

Participants were asked to describe practices regarding social distancing when not providing patient care, PPE use, hand hygiene practices, and exposure to patients with COVID-19 (Table 1). The majority of participants reported wearing a mask while at work (93.2%), washing hands after patient contact (94.6%), and after contact with commonly touched patient and non-patient-care surfaces (95.9%). Eighteen HCP (24.3%) reported contact with COVID-19 patients at least some of the time during the previous six months.

**Table 1.** Practices related to PPE use, infection prevention practices, and exposure to COVID-19 patients among HCP in long-term care settings (n = 74).

Question	Usually/Always N (%)	Sometimes N (%)	Never/Rarely N (%)	Missing N (%)
How often are you able to practice social distancing (staying at least 6 feet apart) while you are at work and are not involved in immediate patient care?	50 (67.6)	16 (21.6)	7 (9.5)	1 (1.4)
In the past **7 days**, how often have you had contact with patients who are known or suspected to have COVID-19 while at work?	1 (1.4)	2 (2.7)	70 (94.6)	1 (1.4)
During the past **6 months**, how often have you had contact with patients who are known or suspected of having COVID-19 while at work?	9 (12.2)	9 (12.2)	54 (73.0)	2 (2.7)
How often do you wear a face mask while you are at work?	69 (93.2)	4 (5.4)	1 (1.4)	0
How often do you wear PPE when taking care of patients who are known or suspected to have COVID-19?	47 (63.5)	1 (1.4)	3 (4.1)	23 (31.1)^a^
How often do you clean your hands after coming in contact with patients or their surroundings?	70 (94.6)	2 (2.7)	1 (1.4)	1 (1.4)
How often do you clean your hands after coming in contact with commonly touched surfaces?	71 (95.9)	3 (4.1)	0	0

Note. PPE, personal protective equipment; COVID-19, Coronavirus disease-19.

a Missing data includes 22 HCP who reported not having taken care of COVID-19 patients.

Of the fifty-one HCP who reported using PPE when taking care of patients suspected or confirmed to have COVID-19, forty-seven (90.4%) reported always using PPE during such encounters. N95 use was reported by 74.5% of HCP, fourteen HCP (27.5%) reported use of surgical/isolation masks, and five (9.8%) reported using a cloth face mask. In addition, use of gloves while caring for patients with COVID-19 was reported by 72.5% of HCP, gowns by 64.7%, face shields by 54.9%, and goggles by 43.1%.

At the time of study enrollment, sixty-four HCP (86.5%) reported having received at least one dose of COVID-19 vaccine, sixty-two of whom (83.8%) had completed a primary vaccine series. Three of the five seropositive individuals were vaccinated.

## Discussion

Our study showed a SARS-CoV-2 anti-N IgG positivity rate of 8.9% (seven of seventy-nine HCP) employed in two long-term care settings in the St. Louis region. Overall seroprevalence was lower than that reported in other studies undertaken among HCP at nursing homes during a comparable time period, including one in the state of Georgia (35.5%), and one in Rhode Island (13.1%).^2,5^ HCP in our cohort reported high rates of workplace PPE availability and use, and high self-reported adherence to infection prevention practices outside the workplace. In addition, a small proportion of HCP (12.2%) had frequent contact with patients suspected to have COVID-19 during the six months prior to study enrollment, and 37.9% of the included HCP did not have direct patient-care roles. These factors may have contributed to the lower seroprevalence in our cohort.

As a comparator to the general population, a targeted sampling and random-digit dialing survey conducted among people residing in St. Louis in October 2020 showed an estimated 5.6% of the general population had a history of infection.^7^ Studies of HCP working in acute care settings have shown that community exposures remain the primary driver of infections and that COVID-19 rates among HCP tend to mirror those of the general population.^8,9^ The same likely holds true for HCP working in long-term care settings included in our cohort. Our study was conducted approximately eight months after the general population survey, and interval infections may explain the higher seroprevalence in this cohort.

We noted serodiscordance among nine HCP who self-reported a prior positive COVID-19 test but had a negative SARS-CoV-2 anti-N IgG test. Absent or waning SARS-CoV-2 antibodies may be contributory to this finding,^10^ especially as all the serodiscordant individuals reported a positive COVID-19 test that occurred more than three months before study participation.

Of note is the high proportion of HCP (67.6%) who reported symptoms potentially consistent with COVID-19 during the six months prior to study enrollment. Only 35.1% of these individuals sought care for their symptoms and just 6% attributed their symptoms to potentially being secondary to COVID-19. This highlights the need for reinforcing symptom recognition for COVID-19 among HCP in long-term care settings.

This study has notable limitations. Sample size was small, and enrollment was limited to two facilities in one geographic region. Given the low positivity rate, we were also not able to perform analyses to determine specific workplace or community risk factors for COVID-19. In addition, all survey data was self-reported and we were not able to verify self-reported prior COVID-19 test results.

Our work shows overall low SARS-CoV-2 seroprevalence in HCP working at long-term care facilities at two facilities in the St. Louis, MO region, one year after the COVID-19 pandemic first impacted this region. Data from this pilot study highlights the need for further studies in this under-studied population, and the continued need to characterize and prevent infectious disease threats in long-term care settings.
